# Airborne Microalgae: Insights, Opportunities, and Challenges

**DOI:** 10.1128/AEM.03333-15

**Published:** 2016-03-21

**Authors:** Sylvie V. M. Tesson, Carsten Ambelas Skjøth, Tina Šantl-Temkiv, Jakob Löndahl

**Affiliations:** a Department of Marine Sciences, University of Gothenburg, Gothenburg, Sweden; b Department of Biology, Lund University, Lund, Sweden; c National Pollen and Aerobiology Research Unit, Institute of Science and the Environment, University of Worcester, Worcester, United Kingdom; d Department of Design Sciences, Lund University, Lund, Sweden; e Stellar Astrophysics Centre, Department of Physics and Astronomy, Aarhus University, Aarhus, Denmark; University of Calgary

## Abstract

Airborne dispersal of microalgae has largely been a blind spot in environmental biological studies because of their low concentration in the atmosphere and the technical limitations in investigating microalgae from air samples. Recent studies show that airborne microalgae can survive air transportation and interact with the environment, possibly influencing their deposition rates. This minireview presents a summary of these studies and traces the possible route, step by step, from established ecosystems to new habitats through air transportation over a variety of geographic scales. Emission, transportation, deposition, and adaptation to atmospheric stress are discussed, as well as the consequences of their dispersal on health and the environment and state-of-the-art techniques to detect and model airborne microalga dispersal. More-detailed studies on the microalga atmospheric cycle, including, for instance, ice nucleation activity and transport simulations, are crucial for improving our understanding of microalga ecology, identifying microalga interactions with the environment, and preventing unwanted contamination events or invasions.

## INTRODUCTION

The presence of microorganisms in the atmosphere has been debated over centuries. Diseases and pest propagation were progressively associated with airborne biological particles (1, 2) composed of a rich microbial diversity of prokaryotic organisms, belonging to archaea and bacteria, including cyanobacteria (e.g., references 3 to 7), and eukaryotic organisms, such as some protozoans, protists, and small metazoans (e.g., references 4 to 8). Yet, it is still unclear to what extent many of our best-known diseases, such as the common flu, are transmitted through the air.

Among airborne microorganisms, microalgae are unicellular photosynthetic organisms whose occurrence has been reported over the last century across a wide range of ecosystems. Due to their small size, a few micrometers to 500 μm (9, 10), microalgae can easily be dispersed by air and water currents and by biotic vectors such as humans and animals. Airborne microalgae are detected in a wide range of ecosystems, at almost all latitudes, from polar to tropical regions (e.g., the Antarctic [11], Central America [12, 13], Europe [5, 14–16], South and Southeast Asia [7, 17, 18], North America [6, 19], and the Central Pacific [20]) (Table 1). They occur in extremely different biomes associated with both organic and inorganic materials (e.g., references 18 and 21) from low altitudes (car level [20]) to high altitudes (troposphere [6, 22]), in dry to wet air samples (e.g., Sahara dust [23] and snow [9, 24]), and over desert to aquatic areas (e.g., references 6 and 17). Airborne microalgae are also frequently monitored indoors, among dust and in biofilm and in sewage disposal (6) and in houses and buildings (14, 18).

**TABLE 1:** List of eukaryotic airborne microalgae^*a*^

Kingdom or subkingdom, phylum or class, and genus	Substrate(s)	Presence in location:										Reference(s)
		Antarctic	South/Southeast Asia	Central America	Europe	Nearctic	Palearctic	Central Pacific	Taiwan	Transatlantic	Eastern USA	
Chromista												
Bacillariophyta												
Achnanthes	Air										x	6
Amphora	Air										x	6
Chaetoceros	Air					x					x	6, 7
Coscinodiscus-like	Air					x					x	6, 7
Cyclotella	Air		x				x					7, 17
Cymbella	Air		x									17
Eunotia	Air		x									17
Fragillaria	Air				x							14, 82
Gomphonema	Air								x			6
Grammatophora	Air				x							8
Hantzschia	Air	x	x		x	x	x		x		x	6, 7, 14, 17, 82
Melosira-like	Air		x			x					x	6, 7, 17
Navicula	Air		x		x	x	x				x	6, 7, 14, 17, 82, 159
Naviculoid diatom	Air								x			6
Nitzschia	Air		x	x	x	x			x		x	6–8, 13, 17, 159
Pinnularia	Air	x	x									7, 17, 159
Stauroneis	Air		x									17
Synedra	Air		x									17
Tabellaria	Air			x								13
Unknown diatom	Air	x	x	x		x	x				x	6, 7, 12
Ochrophyta												
Botrydiopsis	Air					x		x			x	6, 7, 20
Botrydium	Air							x				20
Chromulina	Air										x	6
Chrysocapsa	Air					x					x	6, 7
Haplosiphon	Air							x				20
Heterococcus	Air					x		x			x	6, 7, 20
Heterothrix	Air				x		x					7, 14, 82
Heteropedia	Air					x						7
Monallantus	Air							x			x	14, 20, 82
Monocilia	Air										x	6
Spumella	Air				x							8
Tribonema	Air					x					x	6, 7
Vaucheria	Air					x					x	6, 7
Viridiplantae												
Charophyta												
Closterium	Air		x									159
Coleochaete	Air								x			6
Cosmarium	Air					x					x	6, 7
Cylindrocystis	Air					x		x			x	6, 7, 20
Klebsormidium	Facade				x							15
Mesotaenium	Air			x								12, 13
Mougeotia	Air				x							8
Roya	Air					x					x	6, 7
Zygnema	Air				x			x				7, 8, 20
Chlorophyta												
Actinastrum	Air				x		x					6, 7
Ankistrodesmus	Air					x					x	6, 7
Apatococcus	Air				x							15
Asterococcus	Air					x			x		x	6, 7
Borodinella	Air					x					x	6, 7
Botryokoryne	Air			x								12, 13
Bracteacoccus	Air					x		x	x		x	6, 7, 28
Chaetophoracean-like	Air							x				20
Chlamydomonas	Air	x		x	x	x		x	x		x	6–8, 13, 14, 20, 82
Chlorella^*b*^	Air		x	x	x	x	x	x	x		x	6–8, 12–15, 17, 20, 82, 159–161
Chlorococcum^*b*^	Air		x	x	x	x	x	x	x	x	x	5–7, 12–14, 17, 20, 82, 159
Chlorohormidium	Air				x							7, 14, 82
Chlorosarcina	Air					x		x	x		x	6, 7, 20
Chlorosarcinopsis	Air, facade				x	x		x			x	6–8, 15, 20
Chlorosphaera	Air	x										7
Chlorosphaeropsis	Air					x					x	6, 7
Choricystis	Facade											15
Coccobotrys	Facade				x							15
Coccomyxa	Air, facade				x				x			6, 15
Coelastrum	Air					x					x	6, 7
Desmococcus	Facade				x							15
Dictyochloris	Air					x					x	6, 7
Dictyococcus	Air							x				20
Dimorphococcus	Air	x										7
Diogenes	Air			x								13
Eudorina	Air										x	6
Friedmannia	Air					x					x	6, 7
Geminella	Facade				x							15
Gloeococcus	Air								x			6
Gloeocystis	Air				x	x			x		x	6–8
Hematococcus	Air				x							8
Hormidium	Air	x	x	x	x	x	x	x	x	x	x	5–7, 12, 13, 20, 159, 160
Hormotila	Facade				x							15
Hormotilopsis	Air										x	6
Keratococcus	Facade				x							15
Klebshormotilopsis	Air					x						7
Lobosphaera	Air				x							8
Microspora	Air					x					x	6, 7
Microthamnion	Air							x				20
Monoraphidium	Air				x							8
Myrmecia-like	Air								x			6
Nannochloris	Air					x		x			x	6, 7, 20
Neochloris	Air					x		x			x	6, 7, 20
Oedogonium	Air		x			x					x	6, 7, 159
Oocystis^*b*^	Air		x			x		x	x		x	6, 7, 17, 20
Ourococcus	Air					x					x	6, 7
Palmella	Air					x					x	6, 7
Palmellococcus	Air					x			x		x	6, 7
Palmellopsis	Facade				x							15
Pediastrum	Air						x					7
Planktosphaeria	Air					x			x		x	6, 7
Pleodorina	Air					x						7
Pleurastrum	Air					x					x	6, 7
Prasiola	Air	x				x					x	6, 7
Pleurococcus/Protococcus^*b*^	Air		x		x	x	x		x	x	x	5–7, 14, 17, 82
Protosiphon	Air					x					x	6, 7
Pseudulvella-like	Air					x					x	6, 7
Radiococcus	Air					x					x	6, 7
Radiosphaera	Air					x		x			x	6, 7, 20
Rhizoclonium	Air					x		x	x		x	6, 7, 20
Rhopalocystis	Air	x										7
Scenedesmus^*b*^	Air		x	x	x	x	x	x	x		x	6–8, 12–14, 17, 20, 82
Selenastrum	Air		x									7, 159
Sphaerocystis	Air					x					x	6, 7
Spongiochloris	Air					x					x	6, 7
Spongiococcum	Air					x					x	6, 7
Stichococcus	Air, facade		x		x	x	x	x	x	x	x	5–8, 14, 15, 20, 28, 82, 159, 160
Tetracystis	Air					x		x			x	6, 7, 20, 161
*Tetraëdron*	Air					x					x	6, 7
Tetraspora	Air					x					x	6, 7
Trebouxia	Air, facade				x	x			x		x	6–8, 15
Trentepohlia	Air, facade				x			x	x			6, 15, 20
Treubaria-like	Air										x	6
Ulothrix	Air			x		x					x	6, 7, 12, 13
Westella	Air					x					x	6, 7

a “x” indicates the presence of a taxon at the location. Viable cultures have been established from all locations except for the Transatlantic.

b Culture identified as harmful.

Taxonomically, airborne microalgae belong either to the prokaryotes cyanobacteria (also known as blue-green algae) or to some unicellular microeukaryotes. Genitsaris et al. (8) stated that 353 morphological taxa have so far been monitored in the atmosphere. Cyanobacteria compose a major part of the diversity and have been extensively reviewed in the past few years (8, 21, 25). Among the eukaryotic microalgae, about 114 genera were identified (Table 1). They are principally represented in the atmosphere by the phylum Chlorophyta, commonly called “green algae,” and the genera Chlorella and Chlorococcum (26). On the other hand, the kingdom Chromista is represented by the phyla Bacillariophyta and Ochrophyta in the atmosphere (taxonomic classification from www.algaebase.org).

Little is known about the atmospheric cycle of microalgae, despite their common presence in aerial, aquatic, and terrestrial ecosystems. The different steps of the atmospheric cycle (emission, transport, deposition, and settlement) and the environmental variables that influence it are reviewed in the section Atmospheric Cycle of Airborne Microalgae. Furthermore, information is provided on the causes of airborne microalga deposition and the consequences of their dispersal. Microalgae can, for instance, interact with the surrounding environment during atmospheric transportation and potentially affect, at a larger scale, meteorological events (see the section Consequences of Airborne Microalga Settlement for Health, Economy, and Environment) (also, e.g., reference 27). Once deposited, some microalgae can reproduce, at least somatically, in a new environment (e.g., experiments on agar plates [6, 28] and in water tanks [16]) and potentially cause environmental and sanitary issues (see the section Consequences of Airborne Microalga Settlement for Health, Economy, and Environment). Their capacity for survival over long-distance transportation and in atmospheric microhabitats, their ability to induce their own deposition, and the consequences of their dispersal are still not fully understood.

One reason for the lack of knowledge is that current technology limits ecological investigations of airborne microalgae. First, certain techniques are ineffective in collecting and/or detecting the whole diversity of airborne microalgae, e.g., by omitting rare and small microalgae from air samples (see the section Technical Issues in Collecting and Identifying Airborne Microalgae). Their detection and isolation from air samples are further challenged due to the limited abundance of microalgae in the atmosphere (10^−4^ to 10^4^ cells per m^−3^ [9, 29]) and heterogeneous distributions (7). Their concentration is difficult to estimate among the more abundant nonbiogenic particles or taxa that are more abundant, larger, and cultivable (see Technical Issues in Collecting and Identifying Airborne Microalgae) (2, 30). Second, it has been proposed that microorganisms can use the atmosphere as a transitory habitat (temporal niches concept [3]); yet, these microhabitats are difficult to recreate in the laboratory. Third, tracking airborne microalgae during their atmospheric cycle and over different spatial scales is complex, and with currently available techniques, it involves large uncertainties (models [see the section Technical Issues in Collecting and Identifying Airborne Microalgae]).

In the present minireview, we report the knowledge available on airborne microalgae from emission, transportation, deposition, and settlement; we identify the impact of such transportation on ecosystems and discuss technical limitations and opportunities when assessing airborne microalga dispersal; and we stress that, to understand the causes and consequences of such dispersal, we need to increase multidisciplinary analyses, including biology, ecology, meteorology, and modeling.

## ATMOSPHERIC CYCLE OF AIRBORNE MICROALGAE

### Emission from a source into the atmosphere.

**FIG 1:**
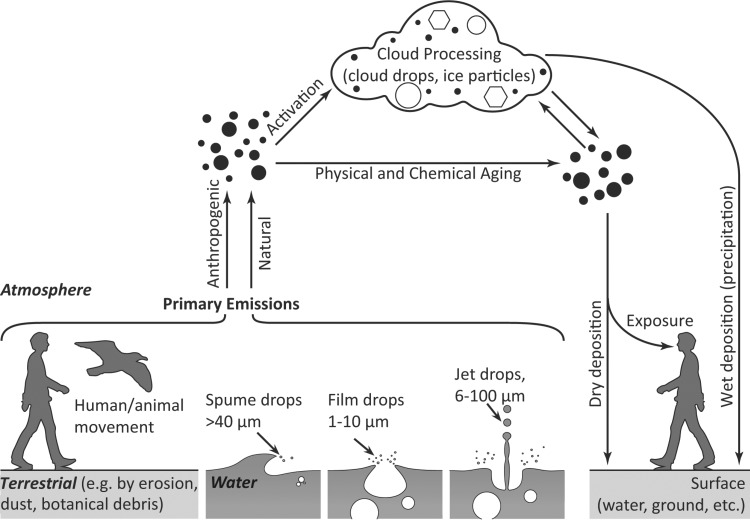
Passive dispersion of airborne particles from emission to deposition (adapted and modified from reference 158 with permission of the publisher).

A range of processes permits the emission of microorganisms into the atmosphere (Fig. 1). Passive processes comprise mechanical erosion by wind and water (e.g., sea spray [31], blown dust [30], and drops [19, 20, 28, 32]) or ecosystem disturbances linked to animal movements and human activities (e.g., reference 18).

Droplet formation is a major factor of passive emission of microorganisms from aqueous ecosystems. Depending on the way that they are generated, different types of drops (i.e., spume, film, or jet drops [Fig. 1]) are ejected. Spume drops of a diameter larger than 40 μm are formed by wind friction, breaking wave crests, at wind speeds exceeding 7 to 11 m s^−1^ (31). Film drops (1- to 10-μm diameter), projected in various directions, and vertically emitted jet drops (6- to 100-μm diameter) are generated from bubble bursting which may occur due to, e.g., waves, rainfall, boat traffic, or supersaturation of gases in the water. Drop formation is therefore a major way for microorganisms to become airborne since the water surface microlayer is enriched with biological material (28, 33, 34). Mayol et al. (29) estimated that several thousand unicellular eukaryotes are emitted on a daily basis per square meter of water over the North Atlantic Ocean, an estimation that varies with the location and wind conditions (e.g., references 8 and 29). Once emitted, airborne turbulent kinetic energy (12) drags the microalgae further up into the atmosphere. For instance, Sassen et al. (27) distinctly identified the presence of microalgae in the troposphere after a period of strong wind (e.g., a hurricane).

Emitted particles, including microalgae, are unequally spread over the air column (35). Their vertical distribution is affected by the distance from the emission source and by the atmospheric structure, in particular, processes in the planetary boundary layer (35, 36). Burrows et al. (30) reported gradients of distribution of propelled biogenic particles. Similarly, modeling back trajectories of airborne bacteria, Zweifel et al. (35) could identify geographic regions as the likely source of airborne biogenic material. These gradients were in both studies characterized by denser concentrations close to the surface of emission. Atmospheric structures, such as fog and clouds, act as barriers of emissions in altitude. Carson and Brown (37) reported, for instance, that fog restricted vertical air currents and facilitated redeposition of aerosolized algae in Hawaii, which caused similar algal composition of the terrestrial source and atmospheric community at a location with prevailing fog.

### Transportation.

Once an organism is emitted into the atmosphere, the residence time of the organism is a balance between attraction forces (predominantly gravitational forces associated with the organism mass) and repulsion forces that retain the organism in the atmosphere (mainly the drag forces associated with the organism size, density, and shape) (2). In still environments, the larger, denser, and more spherical that an organism is, the faster it would sink toward the ground and the shorter its residence time in the atmosphere would be (e.g., reference 29). Attraction and repulsion forces associated with atmospheric perturbations such as wind speed/direction and precipitations further affect the distance of transportation of these particles. Changes in atmospheric pressure can be disregarded in most cases.

Emitted microorganisms can be transported over large distances (kilometers to transhemispheres [2, 3, 38, 39]) for hours to weeks, under favorable meteorological conditions, thanks to their small size and large surface-to-volume ratio (40). Moreover, due to the complexity of the landscape and air mass movement, they circumnavigate faster within a latitudinal band (a few weeks) than toward the poles (a few months) (41). To our knowledge, a first report of long-distance transportation of microalgae was in the Atlantic in the intertropical convergence zone from wind-blown Saharan dust (23). Recently, Mayol et al. (29) estimated that 10% of emitted microbes remain airborne 4 days after emission, allowing a unicellular eukaryote, between 0.5 and 5 μm in size (e.g., some microalgae), to travel an average distance of almost 10,000 km. Carriage over long distances is possible for even larger microorganisms when gravitational settling is the governing removal process (e.g., pollen [42]), causing atmospheric transport of several thousand kilometers (e.g., pollen [43] or pathogens [38]).

The transportation of microbes and seeds may be tempered by environmental barriers (landscape fragmentation [44]) and atmospheric structures (e.g., fog or mist, clouds, and wind corridors). These barriers regulate the altitude and canalize the transportation of biogenic particles. Felicisimo et al. (45) showed, for instance, that wind corridors can passively and directionally transport organisms over long distances (e.g., between continents by transoceanic corridors). Local-scale studies on most bioaerosols are, however, rare, and current knowledge is often based on a few experimental campaigns (e.g., reference 46).

### Deposition.

After emission into the atmosphere and transportation, airborne organisms are removed by dry or wet depositions (Fig. 1). The former is the result of particle settling, impaction, or interception, under the influence of wind speed (turbulence), relative humidity, and temperature. Mayol et al. (29) estimated, for instance, that the dry deposition of unicellular eukaryotes in the North Atlantic Ocean was 9.85 eukaryotes m^−2^ s^−1^. Wet deposition, on the other hand, is the result of particle removal by precipitation (rain or snow) through either in-cloud or below-cloud scavenging. These processes are the main removal mechanism for airborne material in the range of ∼0.1 to 10 μm. Furthermore, wet deposition reduces the exposure time of airborne organisms to atmospheric stress (47) during transportation and, thus, has a positive influence on their viability (48). A major part of global precipitation is initiated by the process of ice formation in clouds, which depends on the presence of ice-nucleating particles, such as mineral dust and bioaerosols, in the atmosphere (49).

Certain airborne microorganisms of 0.2 to 50 μm in diameter with ice nucleation activity (INA) can induce their own wet deposition, which is initiated by an initial formation of an ice particle and its progressive growth in the cloud until the precipitation size is reached (49). These microorganisms belong to the primary biological atmospheric particles (PBAPs), which constitute a major fraction (∼25%) of atmospheric aerosols larger than 0.2 μm (50). Burrows et al. (47) showed that the cloud condensation nucleus (CCN) activity of PBAPs significantly affects their deposition rate, reducing by a factor of 2 their residence time in the atmosphere. More recently, Hoose and Möhler (51) showed that PBAPs are the most potent ice nuclei (IN) currently known, with the capacity to induce ice formation at temperatures between −12°C and −1°C, while mineral dusts nucleate ice below −15°C. This can reduce their residence time in the atmosphere by a factor of 20 (52).

The capacity to facilitate the formation of ice particles, i.e., ice nucleation activity, has been reported in different microorganisms and is associated with specific macromolecules present at the surface of the microorganisms or in their exudates. In pollen, the presence of nonproteinaceous macromolecules on the surface promotes freezing of water (53). In bacteria, the *ina* genes (54) encode the INA proteins responsible for the ice nucleation activity. INA proteins are excreted by bacteria on submicrometer outer membrane vesicles (55) or exported and anchored in the outer membrane of the bacterial cell wall, where they form aggregates that interact with molecules of water (56, 57). Similarly, the fungal INA proteins of the species Fusarium acuminatum of a size of 2.5 to 3 times smaller than bacterial INA proteins are exported to the outer surface of the fungal cell wall, where they are weakly anchored (58). To our knowledge, this is the only ice nucleation protein sequenced so far in Eukarya.

Ice nucleation activity is also induced in larger organisms (e.g., eukaryotes) by epibiotic INA bacteria. Some bacteria promote ice formation (59, 60) in order to damage plant tissues and feed on host-released organic compounds (61, 62), which may result in massive frost damage of crops (reviewed in reference 61). In other cases, the epiphyte-host association is beneficial for the host, which may use the heat released by ice nucleation activity to maintain certain vital regions at an elevated temperature (63).

Certain microalgae have ice nucleation activity (INA), enabling them to form ice crystals in clouds (27) and in aquatic environments (64), but it remains unclear if the microalgae themselves or their epibacteria are responsible for inducing ice nucleation. Certain microalgae from Antarctic soil (65), as well as from seawater, the sea surface microlayer, and fog in marine areas of high primary production (34, 66, 67), cause ice nucleation at high subzero temperatures. This reaction is not universal among microalgae, suggesting a species-specific reaction (68) or an induced reaction from their associated microbiota (e.g., reference 64). In aquatic microalgae, ice nucleation activity is a means to enhance their attachment to ice cover, securing their position in the photic zone. The detection of different INA bacteria on the surface of diatoms or in biofilms containing diatoms (64, 69, 70) suggests that the observed ice nucleation activity of microalgae at temperatures above −12°C may be linked to INA bacterial colonists. These epibacteria would either induce ice nucleation (64) or boost the microalgal ice nucleation activity (71). Other studies, however, showed that ice nucleation can be induced in microalgae by secreted biomolecules. Biomolecules and ice-binding proteins, for instance, are excreted by microalgae and are involved in formation of sea ice (72–76), which is used in cryopreservation (77). Recently, a common marine diatom was found to form submicrometer INA exudates, which are the likely source of abundant biogenic INA particles in the sea surface microlayer (34). Moreover, two recent papers unambiguously report ice nucleation activity in axenic marine diatoms (intact or fragmented) in the temperature range of mineral dust (<−20°C [78, 79]), suggesting that the diatoms themselves actively induce ice nucleation by producing specific macromolecules or that the surface properties of the diatom frustule (i.e., the hard and porous silica cell wall of a diatom) could induce ice nucleation. The solid mineral surface in clay, for instance, has a characteristic density of active sites that induce ice nucleation (80). However, Alpert et al. (79) reported that there was no correlation between the surface area of the diatom frustule and the temperature of ice nucleation, suggesting that ice nucleation activity associated with low temperature and axenic cultures may be induced, as a secondary effect, by biomolecules. In the atmosphere, certain microalgae can contribute indirectly to the formation of wet deposition by the production of CCNs such as sulfate aerosols (dimethyl sulfide [DMS]). Ice nucleation in microalgae has been explored principally on diatoms that represent ca. 17% of eukaryotic airborne microalgae and 5% of total airborne microalgae. This knowledge gap calls for the examination of ice nucleation activity on a broader spectrum of microalgal species, in particular, species associated with wet depositions. Future studies of ice nucleation activity in microalgae will be crucial for both understanding marine contributions to pools of atmospheric IN (30, 34) and estimating the residence times of airborne microalgae and their impact on environments.

### Settlement and survival capacity.

The last phase of the atmospheric cycle is the settlement of the microalgae in a new environment, possibly leading to dispersal (for a definition, see reference 81). During the transportation phase, active or dormant airborne microalgae are exposed to extreme environmental conditions with a high risk of desiccation and oxidative damage and photodamage. Long-distance transport has been reported to reduce the viability of airborne bacterial communities (48), which is likely also the case with airborne microalgae. Despite that, several viable airborne microalgae were reported at different altitudes and over different biomes, e.g., young land masses (20), remote lands (11), building façades (15), water tanks (8), or indoor surfaces (14, 82). Consequences of microalga settlement are reported in the section Consequences of Airborne Microalga Settlement for Health, Economy, and Environment.

Few patterns of colonization have been demonstrated *post hoc* for airborne microalgae. Genitsaris et al. (16), for instance, identified a pattern of colonization in water tanks composed of a first wave of colonization by heterotrophic nanoflagellates followed by the dominance of chlorophytes, principally represented by Chlorella and Scenedesmus genera. Note that these two genera are commonly found in freshwater and are reported in diverse airborne studies over the world.

Airborne microalgae's survival and their efficiency in dispersing are not well understood and require further investigation. For example, it is unclear at which stage of their life cycle microalgae are most efficient at dispersal and coping with environmental stress. Certain microalgae are able to form resistant stages (dormant cells [83]), as well as sheath and mucilage (e.g., in cyanobacteria [84]), or to tolerate drastic environmental conditions (gradient of salinity [85], temperature [86], and humidity [84]). Jewson et al. (87) demonstrated that diatoms are able to rapidly transition between life stages. It is possible that the propelled microalgae can use one of these survival strategies to withstand different phases of their atmospheric cycle or, if propelled as a vegetative cell, transform into another stage of their life cycle during transportation (e.g., pollen [88]). Resting stages are advantageous after deposition, allowing the organism to stay dormant until environmental conditions improve. Moreover, it is important to investigate further the physiological modifications that affect airborne microalgae during their dispersal. Comparisons could be made between genera present in both airborne and aquatic systems, e.g., the genera Nitzschia and Melosira (see Table 1 in reference 89). These sea ice microalgae are able to survive stressful conditions, including extended periods of low light, low temperature, and high salinity. They can produce substances in aquatic systems, such as air bubbles to control buoyancy (see discussion in reference 89) and pigments (e.g., carotenoids in diatoms) to prevent photodamage (90) and cope with desiccation and osmotic stresses in cold environments (91), or cryopreservation substances (extracellular polymeric substances and ice-binding proteins [see “Deposition”]) that may play a key role in their survival in the atmosphere. For example, extracellular-active proteins, encoded by ice-binding protein genes acquired by horizontal gene transfer (92), can freeze the viable organism in brine pockets (72, 73), isolating it from the surrounding environment, while produced exopolymeric substances play important buffering and cryoprotectant roles (93).

This synthesis points toward three major and still-unanswered research questions: (i) how far can a viable microalga be transported, (ii) which proportion of transported microalgae are effectively dispersed, and (iii) which microalgae can nucleate ice in the atmosphere and how.

## CONSEQUENCES OF AIRBORNE MICROALGA SETTLEMENT FOR HEALTH, ECONOMY, AND ENVIRONMENT

### Consequences for human and animal health.

Alive or not, inhaled airborne microalgae are potentially harmful to animals and humans. Their small size facilitates their inhalation and deposition in the respiratory tract. Deposition rate in the respiratory tract can be estimated as the product of the exposure concentration, the inhaled volume, and the deposition probability of the microalgae once inhaled. In normal adult humans, about 300 cells per hour are deposited in the respiratory tract, assuming a concentration of airborne microalgae of 1,000 m^−3^, a breathing volume of 15 m^3^ per day, and a 50% deposition probability (e.g., reference 94). This number is reduced by half at rest (6) and substantially increases during periods of high ventilation (e.g., during exercise) or during periods when atmospheric microalgae are at high concentrations in the air. Wet deposition acts as a vector for the transportation of microorganisms and plays a relevant role for public health (e.g., many INA organisms are pathogens [95]).

Airborne microalgae are recognized as allergens and antigens. They are the cause of severe medical issues, including respiratory allergies (e.g., hay fevers), asthmatic attacks, dermatitis and skin lesions, rhinitis, and disturbances in lymphatic systems or vital organs (e.g., protothecosis [see review in reference 17]). Secondary metabolites produced by certain microalgae are the causes of further human illnesses (e.g., aerosolized algal toxins [96, 97]). The sensitivity of the target can increase when coupled with high temperature or pollutant concentrations (e.g., references 98 and 99), to which these microalgae are resistant (100). Supplemental examples of damage and cytological interactions are available in recent reviews (8, 25).

Are humans and animals safer indoors? There is generally a penetration of atmospheric particles into indoor environments, but the amount is highly variable, as it is determined by factors such as building ventilation systems, human activities, window and door openings, and local climate. Data for microalgae are scant, with only a recent study reporting up to 1.7-times-higher concentrations outdoors (18). Airborne microalgae are preferentially monitored in open and occupied areas. They can easily penetrate indoor environments through available openings (windows, doors, and ventilation systems [e.g., reference 101]) and be spread by animal- and human-mediated movements (soils [84] and movement [18, 102]). Their settlement is promoted by specific environmental conditions identified in different studies as constant, warm, and humid indoor environments with relatively dim light (18, 82, 103).

### Consequences for the environment.

Deposition and subsequent colonization have an impact on the environment. Depending on their ecological strategy, viable deposited microalgae can form seed banks or can proliferate rapidly in a suitable environment (16), colonizing empty niches, increasing community diversity, or supporting the development of organisms in pioneer environments (15, 16, 37). Certain microalgae can be harmful, forming blooms that cause public health, economic, and recreational issues (8, 25, 104, 105). Others are invasive and lead to unexpected biogeographic expansion in freshwater habitats (106). Such colonization affects the community structure, introduces competition between new and resident microorganisms (107), and changes community dynamics (e.g., reference 16).

The settlement of airborne microalgae in aquatic/terrestrial environments constitutes a threat for environmental, economic, and sanitation issues. Harmful and noxious microalgae are able to produce toxins and extracellular compounds that are accumulated in the water column, causing recreational disturbances (e.g., skin irritations or change in the water color) and deteriorating water supplies (e.g., references 108 and 109). Certain toxins can also be accumulated in the food chain, resulting in seafood poisoning and affecting fishery activities (e.g., references 110 and 111).

Airborne microalgae are a factor in building deterioration. Outdoors, green algae and diatoms can develop on walls (21, 112), where they are able to create biofilms, progressively damaging the facades of buildings (15). Their installation is facilitated by the roughness, porosity, and dampness of the material (15, 113, 114). For instance, damp substrate will preferentially be targeted by mucilaginous algae, while small nude unicellular algae prefer to grow on a low-humidity substrate (15). The presence of terrestrial vegetation can also contribute to the deterioration of the material by recruiting certain airborne microalgae (e.g., Choricystis, Chlorella, and Trebouxia) and subsequent waves of colonization by larger organisms (e.g., ferns, moss, and higher plants) (15).

Cloud condensation nuclei (CCNs) and ice nuclei (IN [see “Deposition”]) are able to affect the atmospheric water cycle (115), influencing the development of mixed-phase clouds and affecting global patterns of precipitation (61, 116). Atmospheric ice crystals commonly occur above −8°C. While most mineral dust can induce ice formation only below −15°C (117), microorganisms have a special ability for “heterogenous ice nucleation” at high temperatures (between −1°C and −15°C) (117). Therefore, biological aerosols, being active in the temperature range above −15°C, may drive much of the atmospheric freezing. Locally, high concentrations of ice nuclei modify hydrological cycles, boundary-layer dynamics, cloud lifetime, radiative forcing, and, indirectly, the albedo (30, 118, 119). The phenomenon can be amplified by the nonuniform distribution of the cloudscape across altitude and latitude (inferred from satellite imaging [118]). In Nordic countries, for instance, precipitation almost always occurs by heterogeneous ice nucleation, independently of the concentration of nuclei (120).

## TECHNICAL ISSUES IN COLLECTING AND IDENTIFYING AIRBORNE MICROALGAE

Sample collection and taxonomic identification of airborne microalgae are two major bottlenecks.

The efficiency of an air sampler depends on its inlet, its ability to capture the airborne particles, and, in the case of microbial material, its ability to preserve the relevant biological characteristics such as viability or cell structures (121). The inlet should ideally collect the particles isokinetically (i.e., the air velocity at the inlet should be similar to the surrounding velocity), be of conductive material, and have minimum tubing and bends. At the collection point (e.g., a filter or liquid), an efficient deposition mechanism is needed to capture bioaerosols efficiently. Depending on the air sampler, a significant amount of material may be trapped before reaching the collection point, which reduces the collection efficiency and leads to an underestimate of the concentration.

A range of techniques exists for detection of airborne microorganisms, including microalgae, and for sample collection directly into liquid, on filters, or on agar plates (2). Collection into liquids is most readily made with an impinger, where the air is bubbled through a container of liquid. The collection efficiency is high for airborne particles larger than 1 μm, but a significant fraction may bounce or be reaerosolized by the bubbles (122). Collection on filters may be achieved either by drawing air directly through the filter or by using an impactor. Collection on agar plates could be done directly by an impactor or indirectly by collection on a gelatin membrane filter that is transferred to the agar immediately after sampling. These techniques could be adjusted to get time-resolved sampling as in a rotating slit sampler or tape-band samplers (e.g., reference 2). If viability is an issue, it is important to consider the stress that the sampling imposes on microorganisms. A particular challenge with microalgae is that their concentration in the air is low and therefore methods are needed that sample high volumes of air to get a sufficient amount of material. There are several techniques available for high-volume sampling on filters or agar plates, but high-volume sampling into liquids is more complicated, as high airflows may lead to evaporation and reduce collection efficiency.

Highly time-resolved detection may facilitate understanding of dispersal and transport of airborne microbes. Airborne biological material could be monitored with a time resolution down to seconds with methods based on, for instance, light scattering, fluorescence, mass spectrometry, or flame emission, but further sample analyses are usually necessary to classify the microbial material (123). However, many of the techniques with high time resolution are too unspecific to provide useful information on microalgae, as these constitute a small fraction compared to other particles in the air that, by number, usually are several orders of magnitude more common.

Microscopy (e.g., light microscopy) allows for the estimation of microorganisms' diversity, abundance, cell integrity, and life stages. Cell integrity and vitality can be assessed using permissive techniques such as chemical treatments or microscopic observations after a period of culture from environmental samples or (single-cell-isolated) monocultures. The choice of the medium (e.g., agar plates or liquid medium) and the time in culture make the estimation of microalga biodiversity difficult. Cultivation-based techniques are time-consuming and selective for only a small fraction of organisms that can be grown in the laboratory. Culturing airborne microalgae also selects against organisms that have a long lag phase of growth (e.g., up to 20 days to 10 weeks [18, 124]) and does not permit establishing at which stage of the life cycle (dormant/vegetative cells) a microalga would be transported. Taxonomic identification of microalgae using morphological features may underestimate the genetic diversity present in the samples (e.g., morphospecies and cryptic species). Furthermore, some organisms may be too rare to be detected and properly identified. To refine the identification at smaller scales, more-accurate observations of particular organisms and their microbiome can be performed using a range of electron or differential interference contrast microscopes. These methods are not always commonly available and may be costly and time-consuming, restricting the number of observations.

To overcome identification errors based on morphometric parameters, genetic investigations permit the rapid identification of the taxonomic diversity at different taxonomic levels. Genetic characterization can be performed on cultures, single cells, or environmental samples. A step forward is the use of high-throughput sequencing to rapidly assess microalgal diversity of even very rare community members directly from environmental samples (125). For instance, the v4/v9 regions of the 18S ribosomal DNA are often used in water and soil (126, 127) and in aerosol (bacteria and lichens [128]) samples to assess taxonomic composition and diversity in protists. Due to a high copy number of certain genes (e.g., reference 129), it is important to carefully choose the marker of interest in order to be able to extrapolate the diversity and abundance.

To accurately describe airborne microbial diversity, including microalgae, a combination of high-throughput genetic and microscopy techniques associated with physical and chemical parameters is needed. Such settings would not only permit the identification of diversity and its abundance but also facilitate the investigation of interactomes (e.g., reference 130).

## MODELING THE AIRBORNE MICROALGA ATMOSPHERIC CYCLE AND ITS CONSEQUENCES

Over the last few decades, there has been rapid technical development enabling new possibilities of elucidating the atmospheric cycle of airborne microalgae and their impact on sink environments. Below, we provide an outline of major available models within airborne microbiology with emphasis on microalgae.

To our knowledge, no atmospheric models (Table 2) have been applied to microalgae to totally or partially assess their atmospheric cycle. In a recent paper, Mayol et al. (29) modeled the emission-transportation-deposition of small unicellular eukaryotes in the size range of microalgae. However, the authors did not mention if these protists were photosynthetic organisms, nor did they discuss their taxonomy. Table 2 reports an exhaustive list of atmospheric models that have been used to study specific bioaerosols over a range of spatial scales. These models are considered suitable for studying protists or similar bioaerosols. Further models are also available, such as the EMAC model (131) or TM5 models (132), but were not included in Table 2 because of the lack of taxonomic information.

**TABLE 2:** Atmospheric models for studying sources and transportation of bioaerosols at different spatial scales^*a*^

Model category	Atmospheric model	Model type	Model reference	Bioaerosol(s) (scale[s])	Reference(s)
Receptor model	ACDEP	Trajectory	162	Ragweed (β-α), birch (β-α)	42, 163
	HYSPLIT	Trajectory and particle dispersion	164	Ragweed (α), birch (α-β-γ), oak (β-γ), Alternaria (β-α), pine (γ), Ganoderma (β-γ), olive (β-γ)	43, 134, 136–138, 165–167
	SILAM	Particle dispersion	142	Birch (γ), olive (α)	149, 168
	SGS	Large-eddy simulation	169	Ragweed (μ)	148
	WRF, trajectories	Trajectory	170	Ragweed (β)	171
	ECMWF (172)	Trajectory		Grass (β)	173
Source-based model	OML	Gaussian	146	Grass (μ), ragweed (μ)	136, 151
	SILAM	Eulerian	142	Ragweed (α), birch (α)	174
	METRAS	Eulerian	175	Oak (γ)	88
	DEHM	Eulerian	176	Ragweed (α), birch (α)	177
	KAMM-DRAIS/COSMO-ART	Eulerian	178	Alder (β-α), ragweed (β-α), birch (β-α)	141, 179, 180
	CMAQ	Eulerian	181	Ragweed (β-α), birch (β-α), oak (β-α), grass (β-α), walnut (β-α), mulberry (β-α)	182, 183

a Scales are classified as microscale (μ, 0 to 2 km), meso-gamma (γ, 2 to 20 km), meso-beta (β, 20 to 200 km), and meso-alpha (α, 200 to 2,000 km), as described by Orlanski (144) and modified for air quality modeling (184).

To investigate the transport of airborne particles, including microorganisms, several atmospheric receptor-based and source-based models are available (Table 2). Receptor-based models infer atmospheric transport of particles to/from a randomly selected area, using ground-based or airborne observations, and potentially the history and transport time of these particles (e.g., reference 133). Such model simulations have been applied for different microorganisms, such as bacteria (36), fungal spores (134–136), and pollen (137–140), and can simulate the transport of airborne microalgae. Source-based models require further knowledge about the emission process (location, timing, and amount) over large geographical areas (e.g., reference 141) and consider the turbulence, advection, and deposition properties of the particles (e.g., reference 142). Source-based models are commonly used, for instance, to estimate the concentration of airborne particles at a site (with/without observations) and for large-scale forecasting (e.g., reference 141). However, the main limitation for using source-based models is the uncertainty in the mechanisms of emission of the particles (biological versus physical [141]). Consequently, a larger portion of atmospheric studies on typical bioaerosols such as pollen, bacteria, or fungal spores is based on receptor-based rather than source-based models (Table 2). Additionally, these studies have not considered simulations across spatial scales, mainly focusing on mesoscale applications and often neglecting local-scale simulations (e.g., reference 46).

Modeling transportation over different scales is challenging (46). First, the emission source varies across spatial scales and time (43). Second, the concentration of airborne particles in an air mass is not constant over spatial scale and decreases with increased distance from the source (e.g., by a factor of 10 within the first 100 m [143]). Third, air masses encounter different sources of particles during transport, which affect diversity and air mass footprint. Burrows et al. (30) showed a dominance of dust in air samples collected in the Northern Hemisphere, where continental surfaces are commonly located, and a clearer signal of marine biogenic sources in the Southern Hemisphere. Footprint models can be used to evaluate the direction and distance of transportation of these particles. Last but not least, most atmospheric models are designed for studying transportation of particles at a particular spatial scale (144) (Table 2), while air masses can transport particles over long distances covering several spatial scales (e.g., references 137 and 145). At microscales (0 to 2 km, e.g., sea breeze), particle transportation is typically investigated using Gaussian models (e.g., OML and AERMOD [146, 147]) or large-eddy simulation models (e.g., reference 148). At meso-gamma scale (2 to 20 km), Gaussian models, Eulerian models (88), or trajectory or particle dispersion models (e.g., reference 149) (Table 2) are used, while at larger scales, i.e., meso-beta (20 to 200 km) and meso-alpha (200 to 2,000 km), atmospheric models such as Lagrangian trajectory or particle models or Eulerian models are the main tools (Table 2). Receptor models, including trajectory and particle dispersion models such as HYSPLIT (Table 2), are used over all spatial scales (Table 2) in pollen and smaller particles (e.g., unicellular eukaryotes [29] and green bacteria [150]) and can be the most suitable candidates for studying airborne microalga dispersal. However, an integrative approach using a combination of different models across spatial scales can be used (e.g., reference 151). This could be based on nested strategy, as in air quality modeling (e.g., reference 152), where results from one model feed into another. This approach has recently been proposed as a method to study transportation of microorganisms over different scales (pollen [143]).

Moreover, basically all atmospheric transport models consider microorganisms passive tracers, omitting their capacity of biological transformation (e.g., pollen [88]) and interaction with their environment during transportation (see the section Consequences of Airborne Microalga Settlement for Health, Economy, and Environment). Such biological parameters need to be mathematically formulated and implemented in atmospheric models. Several online models can be used as a complementary tool for receptor-based models. For instance, the impact of INA organisms on their dispersal and meteorological events (e.g., rain and snow) could be further investigated using online weather chemistry models, such as WRF-Chem (153) or COSMO-ART (154), that simulate cloud formation processes over a temporal scale of seconds to minutes (e.g., reference 36).

## CONCLUSIONS AND PERSPECTIVES

The atmospheric cycle of microalgae opens fascinating opportunities for further exploration of microalgal ecology, their adaptation and evolution in the atmosphere, and their interaction with epiphytes for the induction of ice nucleation activity. Following noxious microalgae from their bloom, through the air, toward a new habitat will, for instance, allow prevention of contamination events or invasions. Phytoplankton invasions have already begun and are spreading out of their usual area of occurrence (e.g., reference 106). Presently, it is unclear how these invasive microalgae can disperse so fast (e.g., animal codispersal, human-mediated introduction, or air dispersal) and to what extent environmental parameters favor this acceleration (e.g., temperature [155]). The application of atmospheric models (e.g., Table 2) is a vital but yet unused tool to enhance knowledge. Future environmental scenarios predict an increase in temperature (156) and an alteration of water color (157). It would therefore be important to assess the extent to which these changes will affect the atmospheric cycle and the efficiency of dispersal of airborne microalgae. Further studies also need to investigate the colonization dynamic of airborne microalgae in new habitats and model their transportation to clearly identify possible patterns between source, sink, and risks.
